# Environmental Regulation, Scientific and Technological Innovation, and Industrial Structure Upgrading in the Yellow River Basin, China

**DOI:** 10.3390/ijerph192416597

**Published:** 2022-12-10

**Authors:** Jianshi Wang, Yu Cheng, Chengxin Wang

**Affiliations:** College of Geography and Environment, Shandong Normal University, Jinan 250358, China

**Keywords:** environmental regulation, scientific and technological innovation, industrial structure upgrading, Yellow River Basin

## Abstract

Industrial structure upgrading is an important prerequisite for achieving regional ecological environment protection and high-quality development, and environmental regulation can improve the capacity of scientific and technological innovation and promote industrial structure transformation and upgrading. Based on the panel data of 78 cities in the Yellow River Basin, the relationships among environmental regulation, scientific and technological innovation, and industrial structure upgrading in the Yellow River Basin were analyzed using the mediating effect model and the panel threshold model. The results showed the following findings: (1) Although both formal and informal environmental regulations can promote industrial structure upgrading in the Yellow River Basin, regional heterogeneity and temporal stage characteristics are observed. (2) Transmission mechanism test results show that formal and informal environmental regulations directly affect industrial structure upgrading and indirectly act on it through the level of scientific and technological innovation, showing a significant mediating effect. (3) Taking scientific and technological innovation as the threshold variable, formal environmental regulations have a corresponding relationship with industrial structure upgrading in the form of a broken line, and informal environmental regulations significantly promote industrial structure upgrading after scientific and technological innovation crosses a certain threshold. Finally, we discuss the article and make corresponding suggestions in terms of environmental regulation and technological innovation.

## 1. Introduction

As an important ecological defense, economic zone, and energy resource base in China, the Yellow River Basin holds a crucial position in China’s economic and social development and ecological security [1]. The Yellow River Basin has a long history of development, a good industry foundation, and great market potential [2]. Especially since the reform and opening up, the Yellow River Basin has developed regional economies with distinctive characteristics and achieved success in industrialization and industrial base construction, making it essential for supporting the sustained and stable development of the national economy [3,4]. In the meantime, the economic development of the Yellow River Basin relies mainly on the secondary industry, the industrial development stages within the basin have significant spatial differences, and its overall industrial structure is dominated by labor- and capital-intensive industries. The industrial structures of the provinces in the middle and upper reaches are single and dominated by heavy industries, mainly energy, heavy, and chemical industries, while the proportion of technology-intensive industries is relatively low. In addition, the industrial development model is crude, and the degree of inter-regional industrial homogeneity is high [5,6,7]. Coordinating economic development with environmental protection and fundamentally changing the previous mode of crude economic growth at the expense of the environment have become critical issues that must be addressed to implement ecological protection and high-quality development strategies in the Yellow River Basin. Hence, there is a necessity for comprehensively promoting the industrial structure upgrading and achieving high-quality economic development. Environmental regulations could promote industrial structure upgrading, and technological innovation is an effective approach to promoting industrial structure upgrading [8]. In September 2019, the ecological protection and high-quality development of the Yellow River Basin were established as a major national strategy in China [9]. In this context, clarifying the effects of environmental regulation and scientific and technological innovation on the industrial structure upgrading in the Yellow River Basin can inform government departments to formulate and improve industrial policies and guide industrial structure optimization and upgrading.

Environmental regulation mainly refers to the government’s institutional arrangement to constrain the pollutant discharge behavior of economic entities by promulgating administrative systems, activating market mechanisms, and exercising the role of the public. It aims to address the failure of the market in environmental externalities, intervene in the economic activities of various relevant economic entities, and seek harmonious development of the social economy and the ecological environment [10,11,12]. As an important part of social regulation, environmental regulation can originate from tangible institutions or intangible consciousness [13]. Therefore, environmental regulation can be divided into formal and informal environmental regulation [14,15]. The impact of environmental regulations on industrial structure upgrading has become a research hot spot of widespread concern for scholars worldwide, which has yielded relatively rich research results, mainly including the following two aspects. 

Some studies focused on the direct impact of environmental regulations on industrial structure upgrading. Early studies generally supported the compliance cost theory, which argued that while increasing social welfare, environmental protection necessarily increases the production costs for manufacturers, thus limiting their economic activities and ultimately discouraging industrial structure upgrading [16]. For example, Ramakrishnan et al. studied the relationships among regulations, innovation, and performance in the UK using industrial-sector-level data and concluded that in the short run, environmental regulations had a negative impact on innovation, which in turn negatively impacts the economic performance of the industrial sector [17]. Liu et al. found that when the resource allocation distortion effects of environmental regulations outweigh the externalities, that is, when pollution-intensive industries receive higher benefits from increased factor inputs while accepting the tax penalties, environmental regulations inhibit industrial transformation [18]. Subsequent studies broke away from the original static perspective and began to analyze the relationship between the two from a long-term and dynamic perspective, arguing that good environmental regulations stimulate technological innovation, thereby reducing or completely offsetting the supervision cost and generating an innovation compensation effect that grants enterprises a competitive advantage against international rivals, that is, the innovation compensation theory [19]. Lanoie et al. tested the significance of the different variants of the Porter hypothesis using data on the four main elements of the hypothesized causality chain (environmental policy, research and development, environmental performance, and commercial performance) [20]. Researchers also argued that diverse environmental regulatory policies can accelerate industrial structure adjustment and that the economic incentives and legislative supervision of environmental regulatory policies have a significant positive effect on industrial structure upgrading [21]. However, the different types of environmental regulations and the regional diversity lead to an uncertain relationship between environmental regulation and industrial structure upgrading. For example, Stavropoulos et al. adopted a panel data model to verify the relationship between provincial environmental regulations and industrial competitiveness in China and found that the relationship curve between them is not simply linear but U-shaped [22]. Guan et al. reached a similar conclusion that environmental regulations and industrial structure upgrading have a nonlinear relationship, where the inhibitory effect of environmental regulations on industrial structure upgrading diminishes as the economic development level and human capital level increase [23].

Some other studies focus on the indirect impact of environmental regulations on industrial structure upgrading. Environmental regulations can indirectly impact industrial structure upgrading through multiple paths. Zhang et al. explored the effect of environmental technology standards on enterprise green transformation and the action mechanism using data from the Chinese manufacturing industry from 2000 to 2006, and the results showed that environmental technology standards indirectly contribute to the green transformation of the manufacturing industry through three technological modification mechanisms: terminal governance, capital renewal, and resource restructuring [24]. Hu and Xiong found that environmental regulations indirectly advance the transition toward low-carbon development in China’s industrial sector through energy structure and technological progress [25]. Yu and Lu verified the direct and indirect effects of regional environmental regulations on industrial structure upgrading using a dynamic spatial panel model and panel data of 285 prefecture-level cities in China from 2004 to 2016, and the results showed that regional industrial structure upgrading can be promoted by strengthening the environmental regulations and that regional environmental regulations can effectively alleviate the negative effects of economic development, human capital, and foreign direct investment on industrial structure upgrading [26]. Environmental regulations indirectly affect industrial structure upgrading through a single mechanism. Scholars empirically studied the relationships among environmental regulations, technological innovation, and industrial structure upgrading at the country, province, and city levels, and the results showed that environmental regulations promote industrial structure upgrading mainly by influencing the level of technological innovation, which plays a mediating role between environmental regulations and industrial structure optimization [27,28,29]. However, these studies did not distinguish between the types of environmental regulations, let alone consider the synergistic effects of different environmental regulation types. Research has also shown that environmental regulations indirectly impact industrial structure upgrading through the location selection, scale, and mode of foreign direct investment [30,31]. Yuan and Xiang found that in the long run, environmental regulations increase energy efficiency but hinder labor productivity in the manufacturing industry, thus not providing any favorable support for the “strong” version of the Porter hypothesis [32].

In summary, the existing literature provides some basis for this study but is not free from limitations. First, most of the existing studies are based on national, provincial, or city levels. The ecological protection and high-quality development of the Yellow River Basin have been established as a major strategy for China’s national and regional economic development. Thus, the existing studies lack an examination of the Yellow River Basin. Second, existing studies mostly examine the impact of formal environmental regulations on industrial structure upgrading, thus ignoring the different impacts of informal environmental regulations on industrial structure upgrading. Finally, although some scholars have examined the relationships among environmental regulations, scientific and technological innovation, and industrial structure upgrading, empirical studies integrating formal and informal environmental regulations, technological innovation, and industrial structure upgrading into the same framework are scarce. Based on the above, this study extends the existing research in the following aspects; First, this paper takes 78 cities in 9 provinces/autonomous regions along the Yellow River Basin as the study area to theoretically analyze and empirically test the relationships among environmental regulations, technological innovation, and industrial structure upgrading. Second, the relationships among environmental regulations, technological innovation, and industrial structure upgrading are examined from the perspective of formal and informal environmental regulations. Finally, this study introduces a mediating effect model and a panel threshold model to investigate the mediating effect of technological innovation between environmental regulations and industrial structure upgrading and examine whether environmental regulations have a nonlinear relationship with industrial structure upgrading under different levels of scientific and technological innovation. This study could provide reference and guidance for exploring the effective path by which environmental regulations and scientific and technological innovation synergistically promote industrial structure transformation and upgrading.

The rest of the paper is organized as follows: Section 2 provides a brief analysis of the theoretical foundations and research hypotheses. Section 3 mainly describes the data sources and research methods. Section 4 presents the main findings of the study. Part 5 provides the discussion and conclusion.

## 2. Theoretical Analysis and Research Hypotheses

### 2.1. The Direct Impact of Environmental Regulations on Industrial Structure Upgrading

The direct impact of formal environmental regulations on industrial structure upgrading is as follows: On the one hand, formal environmental regulations promote industrial structure transformation and upgrading through the industrial transfer effect. According to the pollution haven hypothesis, pollution-intensive enterprises often move from countries (regions) with relatively strict environmental regulations to countries (regions) with relatively lax environmental regulations to avoid the compliance costs associated with environmental regulations, thus advancing the industrial structure of their countries (regions) from a lower to a higher stage [8,33,34]. On the other hand, formal environmental regulations promote industrial structure transformation and upgrading mainly through the entry barrier effect. The effects of increased formal regulation intensity include increasing the sunk costs of pollution-intensive enterprises to enter the market, raising the technical requirements for pollutant treatment and discharge, raising the entry barrier for pollution-intensive industries, inhibiting the large-scale expansion of pollution-intensive industries, and promoting the accelerated development of the service sector, thus optimizing the industrial structure [35,36].

The direct impact of informal environmental regulations on industrial structure upgrading is as follows: On the one hand, with increased concern about environmental issues and increased demands for environmental quality due to the promotion of ecological civilization construction, the public often forces pollution-intensive enterprises to reduce environmental pollution and undertake green technological innovation through negotiation and media exposure, thus promoting the clean development of pollution-intensive industries [37,38,39]. On the other hand, the public consumption structure changes with economic development and the increase in residents’ income, and the demand for enjoyment and high-value-added products and services gradually expands. These factors prompt enterprises to improve their existing production processes with new processes and technologies so that the market competitiveness of their products can be improved to meet the consumer demand for high-end products, thus advancing industrial structure transformation [15,40,41]. In the meantime, the effect of informal environmental regulations on industrial structure upgrading is enhanced along with the regional economic development and the increase in residents’ income [42,43].

**Hypothesis**  **1:** *both formal and informal environmental regulations promote industrial structure upgrading in the Yellow River Basin, and the effect of informal environmental regulations on industrial structure upgrading is stronger than that of formal environmental regulations*.

### 2.2. The Indirect Effect of Environmental Regulations on Industrial Structure Upgrading

Formal environmental regulations influence industrial structure upgrading through technological innovation. Based on the Porter hypothesis, the effects of appropriate environmental regulations include stimulating enterprise innovation, increasing innovation capital investment, acquiring advanced technologies, and conducting scientific and technological innovation activities, thus producing the innovation compensation effect and advancing the enterprises toward clean development and high-value-added products [44,45,46]. In the meantime, according to the innovation compensation effect, reasonable environmental regulations can encourage large and medium-sized enterprises to fundamentally reduce compliance costs through technological research and development, green innovation, and providing value-added services. In contrast, small and medium-sized enterprises that cannot afford the high costs of pollution emission and technology development are forced out of the market [47,48,49]. Therefore, during the implementation of environmental regulations, some enterprises enhance their competitiveness and achieve sustainable development through green innovation [50]. Other enterprises that cannot keep up with the green development trend are ruthlessly eliminated by the market, thus achieving the “survival of the fittest” in the process of industrial structure upgrading [51,52,53].

Informal environmental regulations influence industrial structure upgrading through technological innovation. First, the gradually extensive disclosure of environmental information and the increasingly stringent public scrutiny force enterprises to increase their investment in technological innovation, reform their production technology, and improve their raw material utilization out of concern for damage to their reputation and the increased risk of publicized pollution incidents, thus promoting industrial structure optimization and adjustment [54,55,56]. Second, with a strengthened preference for green and environmentally friendly products, consumers are more inclined to buy products with the green certification mark. Therefore, enterprises will increase investment in technological innovation for competitive advantages in return, resulting in environmental technology upgrades. Informal environmental regulatory actions create pressure on enterprises. Pollution-intensive enterprises seeking to maintain their image and achieve long-term development are often forced into technological improvements, ultimately promoting industrial structure upgrading [57,58,59].

**Hypothesis**  **2:** *formal and informal environmental regulations indirectly contribute to the industrial structure upgrading in the Yellow River Basin through the mediator variable of scientific and technological innovation*.

## 3. Model Configuration and Data Sources

### 3.1. Modeling

To test the direct effect of environmental regulations on the industrial structure upgrading in the Yellow River Basin, the following benchmark regression model is constructed:

(1) $$\ln Stur_{it}=\alpha_{0}+\alpha_{1}\ln er_{it}+\alpha_{2}\ln X_{it}+\mu_{i}+\varepsilon_{it}$$

In Equation (1), *Stur_it_* denotes the industrial structure upgrading of city *i* in year *t*; *er_it_* denotes environmental regulations, which are decomposed into formal environmental regulation (Fer) and informal environmental regulation (Ier); *α*_0_ is the intercept term; *α*_1_ and *α*_2_ are the regression coefficients of ln*er* and ln*X*, respectively; *μ_i_* is the individual effect; *ε_it_* is the stochastic perturbation term; and *X* is the control variable.

After the theoretical analysis and the proposal of research hypotheses, the following mediating effect model is constructed to test the mediating role of scientific and technological innovation:

(2) $$\ln Tech_{it}=\gamma_{0}+\gamma_{1}\ln er_{it}+\gamma_{2}\ln X_{it}+\mu_{i}+\varepsilon_{it}$$

(3) $$\ln Stru_{it}=\rho_{0}+\rho_{1}\ln er_{it}+\rho_{2}\ln Tech_{it}+\rho_{3}\ln X_{it}+\mu_{i}+\varepsilon_{it}$$

where ln *Tech_it_* denotes the level of scientific and technological innovation in city *i* in year *t*. The other variables are as described above. Equation (2) tests the impact of formal and informal environmental regulations on scientific and technological innovation, and Equation (3) tests the impact of formal and informal environmental regulations and scientific and technological innovation on industrial structure upgrading. If γ_1_ and ρ_2_ in Equations (2) and (3) are significant at the same time, then scientific and technological innovation is a mediator variable; if *ρ*_1_ in Equation (3) is not significant, then scientific and technological innovation is a full mediator variable.

The panel threshold model is adopted to further analyze the path by which scientific and technological innovation, the mediator variable, affects the effects of formal and informal environmental regulations on industrial structure upgrading in the Yellow River Basin. Specifically, scientific and technological innovation is used as a threshold variable to study its moderating role via which formal and informal environmental regulations affect industrial structure upgrading in the Yellow River Basin. Based on the panel threshold effects, the model is specified as follows:

(4) $$Stru_{it}=\alpha_{i}+\beta_{1}FerI(Tech_{it}\leq \lambda )+\beta_{2}Fer_{it}I(Tech_{it}>\lambda )+\theta_{0}X_{it}+\varepsilon_{it}$$

(5) $$Stru_{it}=\alpha_{i}+\beta_{1}IerI(Tech_{it}\leq \lambda )+\beta_{2}Ier_{it}I(Tech_{it}>\lambda )+\theta_{0}X_{it}+\varepsilon_{it}$$

### 3.2. Variables

#### 3.2.1. Explained Variables

Industrial structure upgrading (*Stru*). The value-added proportions of the tertiary and secondary industries in prefecture-level cities are used to measure the industrial structure upgrading in the Yellow River Basin. If such proportions are on the rise, the economy of the Yellow River Basin is advancing towards servitization, and the industrial structure is upgrading [22].

#### 3.2.2. Explanatory Variables

Formal environmental regulations (Fer). It is particularly important to accurately measure or evaluate the level of environmental regulations in a region and thus select and implement appropriate environmental regulation tools [60]. Scholars usually measure the environmental regulations of a region using indicators such as the number of environmental regulation policies, the investment cost of pollution control, the pollution control effect of environmental regulations, and the per capita GDP [61,62]. To better illustrate the effects of environmental regulations in the Yellow River Basin, we adopted an aggregative indicator of environmental regulation intensity based on the per-unit industrial wastewater emission intensity, per-unit industrial SO_2_ emission intensity, and per-unit industrial fume (dust) emission intensity calculated with the entropy weight method.

Informal environmental regulations (Ier). Based on a previous calculation method, the level of education, per capita income, and population density were selected to measure the informal environmental regulation intensity [14]. Generally speaking, higher levels of education coincide with higher environmental awareness and greater concern about environmental issues. Given the availability of data, the level of education was characterized using the ratio of full-time teachers in institutions of higher learning, general secondary schools, and elementary schools to the total population at the end of the year. Residents with higher incomes often have stronger demands for high environmental quality and higher initiative to promote the green transformation of the industrial structure. In this study, the average wage of employees on the job was used to characterize the per capita income level. With higher population densities, more people are troubled by environmental pollution. Thus, more people will be involved in environmental quality improvement and environmental protection. In this study, the total population at the end of the year and the population per unit area were used to characterize the population density [59,63].

#### 3.2.3. Mediating Variables

Scientific and technological innovation (Tech). Given the availability and continuity of data, the level of scientific and technological innovation for the year was measured by the logarithm of the total number of invention patents, utility model patents, and design patents in prefecture-level cities [47,64].

#### 3.2.4. Control Variables

Industrial structure upgrading is the result of multiple factors, such as environmental regulation and scientific and technological innovation, and is closely associated with factors such as economic development, fixed asset investment, opening up, urbanization, and consumption structure [65,66]. Therefore, to control the influence of other variables on the estimation results and for the availability and comparability of data on the Yellow River Basin, we used economic development, fixed asset investment, and opening up as control variables [67,68]. Specifically, the level of economic development (Rgdp). Economic development promotes industrial structure upgrading by changing the resident consumption structure, advancing the gradual upgrading of consumer goods from low grade to high grade, and driving the development of services, tourism, finance, and other related industries. In this study, the per capita GDP is used to measure the level of economic development. The level of fixed asset investment (IE). Other than driving economic growth, investments expand the scale of market capital supply and affect the existing industrial structure. The level of fixed asset investment is measured by the proportion of fixed asset investment volume to the regional GDP. The level of opening up (Open). The trade level of a country or region acts to industrial structure transformation through capital accumulation, technology spillover, and institutional innovation. The level of opening up was characterized by the proportion of total imports and exports of prefecture-level cities in the regional GDP.

### 3.3. Data Sources and Descriptive Statistics

The Yellow River Basin includes nine provinces (municipalities and autonomous regions), of which Sichuan Province was included in the Yangtze River Economic Belt Development Strategy in September 2016 and became part of the Yangtze River Economic Belt. The four cities (leagues) in eastern Inner Mongolia have close economic and trade ties with Northeast China and have been included in the Northeast Regional Revitalization Plan. Therefore, this study focused on eight provinces (autonomous regions) in the Yellow River Basin, including Qinghai, Gansu, Ningxia, Inner Mongolia (except for its eastern part), Shanxi, Shaanxi, Henan, and Shandong. We calculated the environmental regulation intensity based on the per-unit industrial wastewater emission intensity, per-unit industrial SO2 emission intensity, and per-unit industrial fume (dust) emission intensity, and relevant pollutant data will not be counted after 2018. Due to the serious missing data of some cities, 78 cities in the eight provinces (autonomous regions) were finally selected for the study considering the completeness and accessibility of data. The study period was from 2004 to 2018. Additionally, according to the research needs, the eight provinces (autonomous regions) along the Yellow River Basin were divided into the upper reach region (Qinghai, Gansu, and Ningxia), the middle reach region (Shanxi, Shaanxi, and Inner Mongolia), and the lower reach region (Shandong and Henan). The data used in the study are mainly from the China City Statistical Yearbook, China Statistical Yearbook on Environment, China National Intellectual Property Administration, EPS database, and statistical bulletins of national economic and social development of the relevant cities. The missing values of some indicators are completed through interpolation and other methods. The relevant descriptive statistics for each variable are shown in Table 1.

Based on the above benchmark regression model, the logarithms of the variables were used to ensure the smoothness of the panel data time series and reduce the effect of heteroscedasticity. To avoid problems such as multicollinearity and spurious regressions, the LLC and IPS unit root tests were conducted (Table 2). According to the estimation results, all explanatory variables pass both unit root tests, indicating that the panel data are smooth and can be subjected to subsequent regression analysis.

## 4. Empirical Tests and Result Analysis

### 4.1. Holistic Regression Analysis

The benchmark regression results of environmental regulations on the industrial structure upgrading in cities of the Yellow River Basin are presented in Table 3. Model (1) is based on a fixed effects model and examines the effect of formal environmental regulations on the industrial structure upgrading in the Yellow River Basin. According to the results, the regression coefficient of formal environmental regulations is 0.02 and passes the 1% significance test, indicating that formal environmental regulations have a positive contribution to the industrial structure upgrading in the Yellow River Basin. Model (2) is based on a fixed effects model and examines the effect of informal environmental regulations on the industrial structure upgrading in the Yellow River Basin. According to the results, the regression coefficient of informal environmental regulations is 0.63 and passes the 1% significance test, indicating that compared with formal environmental regulations, informal environmental regulations significantly promote the industrial structure transformation in the Yellow River Basin. With increasingly serious regional environmental problems, the public, as the most direct victims, often express their demand for high environmental quality via “informal regulations” without complex administrative procedures. Thus, informal environmental regulations, as an invisible “barrier”, are becoming increasingly important for the industrial structure upgrading and transformation in the Yellow River Basin.

The differences in resource endowments are large, and the economic development is uneven in the upper, middle, and lower reaches of the Yellow River Basin. In the meantime, the impact of environmental regulations on the industrial structure upgrading in different regions also varies. Therefore, the fixed effects model was used to explore the impact of environmental regulations on industrial structure upgrading in different regions. Models (3) and (5) show the effects of formal environmental regulations on the industrial structure upgrading in cities of the upper and middle reaches and cities of the lower reach, respectively. The results show that the regression coefficients of formal environmental regulations in cities of the upper and middle reaches and cities of the lower reach are 0.02 and 0.01, respectively, which pass the 5% and 10% significance tests. Therefore, formal environmental regulations can promote industrial structure upgrading in cities of the upper and middle reaches and cities of the lower reach. However, the effect of formal environmental regulations on industrial structure upgrading is stronger in cities of the upper and middle reaches than in cities of the lower reach. The main reason is that the Yellow River Basin, especially the upper and middle reaches, is an important base of energy, heavy, and chemical industries with great coal consumption, where industrial development has long relied on power, steel, coal chemistry, nonferrous metallurgy, and other resource-intensive industries, and the proportion of heavy industries is high. After long-term, large-scale, high-intensity energy extraction and industrialization, the resource and environmental constraints are tightening, the regional development is out of balance, and the resources are approaching depletion. Thus, the more stringent formal environmental regulations are more likely to promote industrial structure upgrading in cities of the upper and middle reaches than in cities of the lower reach. Models (4) and (6) show the effects of informal environmental regulations on the industrial structure upgrading in cities of the upper and middle reaches and cities of the lower reach, respectively. The results show that the regression coefficients of informal environmental regulations in cities of the upper and middle reaches and cities of the lower reach are 0.77 and 0.48, respectively, both passing the 1% significance level test, indicating that informal environmental regulations can promote the industrial structure upgrading in cities of the upper and middle reaches and cities of the lower reach.

Since 2012, China has attached unprecedented attention to environmental issues and included ecological civilization in the Report to the 18th National Congress of the Communist Party of China. Meanwhile, considering the dynamic path-dependent effect of regional industrial structure adjustment, the 2004 to 2018 period is divided into two, namely, 2004 to 2012 and 2013 to 2018, to further analyze the period heterogeneity effect of environmental regulations on industrial structure upgrading. Models (7) and (9) show the effects of formal environmental regulations on the industrial structure upgrading in the 2004 to 2012 period and the 2013 to 2018 period, respectively. The results show that the effect of formal environmental regulations on industrial structure upgrading is weaker in the former time period than that in the latter time period, and the significance test is not passed. Since 2013, the Chinese government has formulated, promulgated, and revised a series of laws and regulations on environmental protection. As a result, the intensity of formal environmental regulations has been increasing, which has promoted sustainable economic and social development and industrial structure optimization in the Yellow River Basin. Models (8) and (10) show the effects of informal environmental regulations on the industrial structure upgrading in cities of the Yellow River Basin in the 2004 to 2012 period and the 2013 to 2018 period, respectively. The results show that the effect of informal environmental regulations on the industrial structure upgrading in the Yellow River Basin is significantly positive during the study period, indicating that the public demand for better environmental quality is becoming increasingly urgent as environmental pollution brings more and more negative impacts to life. In the meantime, the rapid development of media and network industries increased the number of nongovernmental environmental organizations and further released the power of nongovernmental environmental protection. As a result, the intensity of informal environmental regulations gradually increased, which promoted the industrial structure upgrading and the industrial structure transformation from pollution intensive to low carbon.

As for the control variables, the coefficient of economic development is significantly positive, indicating that high-quality economic development and the improvement of public living standards can effectively promote the industrial structure upgrading in the Yellow River Basin. Economic development optimizes the allocation of production factors, such as capital, technology, and labor, and drives industrial structure upgrading. The regression coefficient of the effect of fixed asset investment on industrial structure upgrading is significantly negative, indicating that the increase in fixed asset investment is not conducive to industrial structure upgrading in the Yellow River Basin. The probable reason is that fixed asset investment in the Yellow River Basin is mainly in coal, metallurgy, building materials, infrastructure, iron and steel, nonferrous metals, and other resource-intensive industries. The development of these industries is often at the cost of high energy consumption and an overdraft on the ecological environment. Thus, the productivity utilization is low, the resource waste is high, and the pollution emissions are intensive, which is not conducive to industrial structure upgrading. The regression coefficient of the effect of the opening-up level on industrial structure upgrading is significantly positive, indicating that the increase in the level of opening up is conducive to optimizing the industrial structure of the Yellow River Basin. Increased levels of opening up attract high-quality foreign investment and bring advanced production technologies and management experiences, thus improving the production factor allocation efficiency and industrial structure optimization in the Yellow River Basin.

### 4.2. Analysis of the Mediating Effect of Scientific and Technological Innovation

The mediating effect of scientific and technological innovation between formal environmental regulations and industrial structure upgrading is examined in Table 4. The estimation results in the first column show the existence of an aggregate effect of formal environmental regulations on industrial structure upgrading. The estimation results of model (2) show that the regression coefficient of the effect of formal environmental regulations is significantly positive, indicating that formal environmental regulations can significantly improve the scientific and technological innovation capacity of the Yellow River Basin. The estimation results of model (3) show that the regression coefficient of the effect of scientific and technological innovation is positive and passes the 1% significance test, and the regression coefficient of the effect of formal environmental regulations is significantly positive at the 1% level, indicating the existence of a mediating effect. That is, formal environmental regulations can promote industrial structure upgrading in the Yellow River Basin through the mediator variable of scientific and technological innovation, indicating that national innovation strategies and regional innovation policies have relatively good effects in promoting industrial structure upgrading. The strengthening of formal environmental regulations can motivate pollution-intensive industries into improving their scientific and technological innovation capabilities with more resources and transforming and upgrading end-processing technologies and clean production technologies, thus generating the “innovation compensation effect” that improves resource utilization efficiency and promotes the industrial structure upgrading. Models (4) to (6) show the estimation results of the mediating effect of scientific and technological innovation between informal environmental regulations and industrial structure upgrading. The estimation results show that, like formal environmental regulations, informal environmental regulations have an aggregate effect on industrial structure upgrading, and the mediating effect of scientific and technological innovation through which informal environmental regulations promote industrial structure upgrading in the Yellow River Basin is significant.

### 4.3. Panel Threshold Effect Regression Analysis

The threshold effect is tested. Using scientific and technological innovation as the threshold variable and Stata 15.0 as the statistical software, the threshold effect test results and the panel threshold effect regression analysis results are obtained by repeatedly sampling 1000 times using the bootstrap sampling method.

As shown in Table 5, the F-statistics of the single-threshold effect and double-threshold effect tests of Fer are 72.47 and 31.26, respectively, and the corresponding bootstrap *p*-values are 0.002 and 0.020, respectively, which pass the 1% and 5% confidence tests. The F-statistics of the triple-threshold effect is 17.67, and the corresponding bootstrap *p*-value is 0.786, which fails the significance test, indicating that the triple-threshold effect is not significant. Therefore, formal environmental regulations have a double-threshold effect. The single-threshold value is 6.9735, and the double-threshold value is 7.4483. The F-statistic of the single-threshold effect of Ier is 43.09, and the corresponding bootstrap *p*-value is 0.010, passing the 5% confidence test. The F-statistic of the double-threshold effect is 18.32, and the corresponding bootstrap *p*-value is 0.1906, which fails the significance test, indicating that the double-threshold effect is not significant. Therefore, informal environmental regulations have a single-threshold effect, and the single-threshold value is 7.4483.

Table 6 shows the panel threshold model regression results. The threshold effect between formal environmental regulations and industrial structure upgrading in the Yellow River Basin is analyzed first. Using formal environmental regulations as the core explanatory variable, it has a double-threshold effect on industrial structure upgrading with threshold values of 6.9735 and 7.4483, respectively. When scientific and technological innovation is below 6.9735, the regression coefficient of the effect of formal environmental regulations is −0.03 and passes the 1% confidence test, indicating that formal environmental regulations hinder industrial structure upgrading in this interval. When scientific and technological innovation is within the [6.9735, 7.4483] interval, formal environmental regulations have a positive but insignificant effect on industrial structure upgrading. When scientific and technological innovation is above 7.4483, the enhanced intensity of formal environmental regulations can significantly promote the industrial structure upgrading in the Yellow River Basin; that is, formal environmental regulations have a corresponding relationship with industrial structure upgrading in the form of a broken line with two thresholds. Therefore, enterprises have higher productivity and increased profits only when the level of enterprise independent innovation ability is continuously improved, and the more stringent regional environmental regulations have a more significant motivating effect on innovation. At this time, the technical compensation effect of environmental regulations gradually exceeds their cost, and their promotion of industrial structure upgrading is enhanced.

Then, the threshold effect between informal environmental regulations and industrial structure upgrading in the Yellow River Basin is analyzed. Using informal environmental regulations as the core explanatory variable, it has a single-threshold effect on industrial structure upgrading with a threshold value of 7.4483. When scientific and technological innovation is below 7.4483, the regression coefficient of the effect of informal environmental regulations on industrial structure upgrading is 0.46 and passes the 1% significance test, indicating that informal environmental regulations can promote regional industrial structure upgrading when the level of scientific and technological innovation is low. When scientific and technological innovation is above 7.4483, the regression coefficient of the effect of informal environmental regulations on industrial structure upgrading is 0.55 and passes the 1% significance test, indicating that the effect of informal environmental regulations on industrial structure upgrading in the Yellow River Basin is significantly enhanced with the increase in the scientific and technological innovation level. To meet the public’s consumption demand for green products and services, enterprises will increase their investment of capital and efforts in the research and development of green products. Thus, green technological innovation capabilities are enhanced, and the proportion of green industries in the overall national economy is increased, which forces the upgrading of the industrial structure.

## 5. Discussion

Based on the panel, our study found that both formal and informal environmental regulations could promote industrial structural upgrading, which was consistent with existing research findings [69,70,71]. In addition, we found that after science and technology innovation crosses a certain threshold, the interaction between informal environmental regulation and science and technology innovation is further enhanced, thus indirectly affecting the transformation and upgrading of industrial structure. Xie et al. and Li et al. argued that when the level of scientific and technological innovation in a region is enhanced, it will increase the conversion rate of scientific and technological achievements, prompting more production technologies and green technologies to be put into use to meet consumers’ preference for cleaner products and promote green transformation and upgrading of the industrial structure [72,73].

Our findings help to raise the awareness of policy makers to improve industrial structure upgrading, to improve the scientific accuracy and relevance of environmental regulation policies and technology innovation policies, and to alleviate industrial structure upgrading challenges and achieve low carbon development, as well as to strengthen the interaction between environmental regulation and technological innovation and leverage technological innovation. Both formal environmental regulation, mainly by the government, and informal environmental regulation, mainly by citizens and others, can force manufacturers to strengthen technological innovation and support the development of energy-saving and high-tech industries. Technological innovation is an effective transmission path for environmental regulations to be used for industrial structure upgrading, and the government should encourage and support enterprises to carry out technological R&D activities and promote the transformation of innovation results into actual productivity.

While this study systematically analyzes the impact of environmental regulation and scientific technological innovation on industrial structure upgrading, some limitations remain to be addressed in the future. First, due to the limitations of data collection, this paper period selected is from 2004 to 2018. Therefore, future research will focus on collecting multiple measures through multisource data in order to establish a more multidimensional and complete evaluation system for the dynamic assessment of industrial structure upgrading in the Yellow River Basin over a long period of time. Second, the influence of spatial spillovers was not considered when selecting the model [74]. As a public good, environmental regulation has a remarkable cross-regional externality; that is, the regulatory policy in one region will always affect the welfare of another region, so the industrial structure upgrading effect of environmental regulation may also have a spatial spillover effect [75]. In the future, a spatial econometric model can be used for analysis to explore the spatial spillover effect of environmental regulation on industrial structure upgrading.

## 6. Conclusions

Based on the panel data of 78 cities along the Yellow River Basin from 2004 to 2018, a comprehensive investigation of the environmental regulations, scientific and technological innovation, and industrial structure upgrading in the Yellow River Basin was conducted using the panel fixed effects model, mediating effect model, and panel threshold model, and the following conclusions were drawn: (1) Benchmark regression results showed that both formal and informal environmental regulations effectively promoted the industrial structure transformation and upgrading in cities of the Yellow River Basin. In addition, the effect of informal environmental regulations on industrial structure upgrading was stronger than that of formal environmental regulations, and the regional heterogeneity and temporal stage characteristics were obvious. (2) Both formal and informal environmental regulations affected the industrial structure upgrading in the Yellow River Basin through the mediation of scientific and technological innovation, and the mediating effect was significant. The panel threshold regression results showed that with scientific and technological innovation as the threshold variable, formal environmental regulations have a corresponding relationship with industrial structure upgrading in the Yellow River Basin in the form of a broken line, and the promotion effect of informal environmental regulations on industrial structure upgrading was significantly enhanced after the scientific and technological innovation crossed a certain threshold.

This study considered the current industrial structure upgrading trends in the Yellow River Basin and examined the direct effects of formal and informal environmental regulations on industrial structure upgrading in the Yellow River Basin and the indirect effects on industrial structure upgrading through the mediation of scientific and technological innovation.

(1) Reasonably increasing the intensity of formal environmental regulations could promote industrial structure transformation and upgrading. The government should strengthen environmental regulations, appropriately increase the intensity of formal environmental regulations, and restrain the pollution emission behaviors of enterprises. In this way, industrial structure optimization can be promoted with environmental regulations while solving environmental problems. Considering the significant regional differences in geographic conditions, resource endowment, industrial structure, and economic development in the Yellow River Basin, it is important to avoid the “one-size-fits-all” approach in the formulation and implementation of environmental regulation policies. Instead, the government should control the strength and timing of formal environmental regulations according to local conditions and seasonal conditions.
(2) A diversified environmental governance system must be constructed, and the role of informal environmental regulations in promoting industrial structure upgrading must be emphasized and strengthened. The per capita income level should be raised and synchronized with economic growth. In the meantime, investment in education should be increased, and the level of human capital should be raised to enhance the public’s awareness and ability to act on environmental protection. The government should take measures, such as strengthening environmental protection education and publicity, actively disclosing enterprise information related to environmental protection, improving the environmental protection reporting and petitioning systems, establishing green channels for environmental protection, and rewarding people related to environmental protection and reporting pollution to guide the public to actively participate in environmental protection.
(3) In environmental regulation and industrial structure upgrading, maximize the conduction effect of technological innovation. It is important to recognize how science and technology innovation can positively influence formal environmental regulation and industrial structure change, and to avoid squeezing out investment in science and technology innovation while increasing environmental management, and to guide more capital to high-tech industries. By promoting environmental regulation and science and technology innovation, we will promote industrial structure upgrading synergistically, and we will encourage enterprises to use technological innovation as a driving force for transforming traditional polluting industries into high-end, intelligent, and green industries. Developing eco-economy, circular economy, and digital economy as new industries and business models to develop a modern industrial system and upgrade the industrial structure.
(4) The government should build a green scientific and technological innovation system and stimulate the vitality of enterprises’ independent research and development. The government should stimulate enterprises’ research and development enthusiasm and improve their independent innovation capability through policies such as financial subsidies, scientific and technological support, patent protection, and perfecting the science and technology investment and financing system. The government should promote the flow of production factors such as capital and talents to science and technology innovation in order to optimize the allocation of production factors and promote the transformation of scientific and technological achievements. The government should also focus on the transformation and upgrading of traditional industries and the development needs of strategic and emerging industries to help the transformation of resource-intensive industries in the Yellow River Basin to technology-intensive industries and clean and environmentally friendly industries, thus promoting the industrial structure optimization and upgrading.

## Figures and Tables

**Table 1:** Descriptive statistics for variables.

Variable	Obs	Mean	Std.dev	Min	Max
lnStru	1170	−6.327	1.7320	−13.050	−0.220
lnFer	1170	−2.442	0.398	−3.585	−0.252
lnIer	1170	−0.356	0.496	−2.004	2.249
lnRgdp	1170	10.210	0.806	7.662	12.460
lnIE	1170	−3.327	1.695	−10.390	0.377
lnOpen	1170	−0.383	0.395	−2.439	0.787
lnTech	1170	6.1250	1.756	0.000	10.580

**Table 2:** Panel stability results.

Variable	LLC	*p*-Value	IPS	*p*-Value
lnStru	−8.1778	0.000	−4.3670	0.000
lnFer	−11.2128	0.000	−15.2986	0.000
lnIer	−8.0534	0.000	−7.9504	0.000
lnRgdp	−13.3708	0.000	−5.4530	0.000
lnIE	−5.6688	0.000	−4.1160	0.000
lnOpen	−8.6561	0.000	−3.3612	0.000
lnTech	−6.6736	0.000	−2.3891	0.008

**Table 3:** Benchmark regression results of environmental regulation on industrial structure upgrading.

Variable	All Observations		Cities of the Upper and Middle Reaches		Cities of the Lower Reach		2004–2012		2013–2018	
	(1)	(2)	(3)	(4)	(5)	(6)	(7)	(8)	(9)	(10)
lnFer	0.02 ***		0.02 **		0.01 *		0.01		0.04 ***	
	(3.61)		(2.37)		(1.85)		(0.18)		(4.08)	
lnIer		0.63 ***		0.77 ***		0.48 ***		0.10 *		0.80 ***
		(13.30)		(11.25)		(8.03)		(1.79)		(8.76)
lnRgdp	0.07 ***	0.26 ***	0.01	0.42 ***	0.18 ***	−0.03	0.04 **	−0.10 ***	0.06	−0.06
	(3.94)	(8.74)	(0.42)	(9.30)	(6.99)	(−0.97)	(2.57)	(−2.83)	(1.09)	(−1.20)
lnIE	−0.04 ***	−0.04 ***	−0.07 ***	−0.06 ***	−0.02	−0.025 *	−0.01	−0.01	−0.01	−0.01
	(−3.86)	(−4.06)	(−4.35)	(−3.90)	(−1.35)	(−1.74)	(−0.68)	(−0.77)	(−0.45)	(−0.65)
lnOpen	0.13 ***	0.07 ***	0.09 **	0.02	0.220 ***	0.132 ***	−0.04 *	−0.05 *	0.05	0.09
	(4.44)	(2.64)	(2.21)	(0.62)	(4.67)	(2.90)	(−1.69)	(−1.88)	(0.83)	(1.46)
Constant	1.08 ***	3.78 ***	−0.48 *	5.75 ***	2.276 ***	1.004 **	−0.08	0.72	0.58	2.14 ***
	(4.98)	(9.04)	(−1.67)	(9.22)	(7.44)	(1.97)	(−0.41)	(1.49)	(0.97)	(3.26)
N	1170	1170	675	675	495	495	702	702	468	468

Note: ***, **, and * indicate significance at the 1%, 5%, and 10% levels, respectively, here and below.

**Table 4:** Regression results of mediating the effect.

Variable	(1)	(2)	(3)	(4)	(5)	(6)
lnFer	0.02 ***	0.04 ***	0.02 ***			
	(3.61)	(2.90)	(2.83)			
lnIer				0.63 ***	1.70 ***	0.47 ***
				(13.30)	(19.45)	(8.32)
lnTech			0.17 ***			0.01 ***
			(11.08)			(5.84)
Constant	−1.08 ***	−8.28 ***	0.29 ***	3.79 ***	5.01 ***	3.33 ***
	(−4.98)	(−19.86)	(1.22)	(9.04)	(6.41)	(7.48)
Controls	Yes	Yes	Yes	Yes	Yes	Yes
N	1170	1170	1170	1170	1170	1170

Note: *** indicates significance at the 1%.

**Table 5:** Threshold regression test results.

Core Explanatory Variable	Threshold Variable	Threshold of the Order	F	*p*	100%	50%	1%
Fer	Tech	First-order threshold	72.47	0.002	35.6829	40.1097	56.1557
Fer	Tech	Second-order threshold	31.26	0.020	20.8391	24.6757	38.7355
Fer	Tech	Third-order threshold	17.67	0.786	48.9425	54.9072	69.1885
Ier	Tech	First-order threshold	43.09	0.010	26.2386	30.8827	40.9297
Ier	Tech	Second-order threshold	18.32	0.196	22.5147	25.4353	31.5795
Ier	Tech	Third-order threshold	9.07	0.794	36.5958	45.2787	61.8908

**Table 6:** Panel threshold regression results.

Variable	Core Explanatory Variable Fer	Core Explanatory Variable Ier
Fer (lnTech ≤ 6.9735)	−0.03 ***	
	(−3.87)	
Fer (6.9735 < lnTech ≤ 7.4483)	0.01	
	(0.16)	
Fer (lnTech > 7.4483)	0.03 ***	
	(4.59)	
Ier (lnTech ≤ 7.4483)		0.46 ***
		(8.13)
Ier (lnTech > 7.4483)		0.55 ***
		(10.50)
Constant	−0.05 ***	3.73 ***
	(−0.24)	(8.60)
Controls	Yes	Yes
N	1170	1170

Note: *** indicates significance at the 1%.

## Data Availability

The data presented in this study are available on request from the corresponding author.

## References

[1] Guo S., Ma H. (2021). Does industrial agglomeration promote high-quality development of the Yellow River Basin in China? Empirical test from the moderating effect of environmental regulation. Growth Chang..

[2] Zhang S., Zhang G., Ju H. (2020). The spatial pattern and influencing factors of tourism development in the Yellow River Basin of China. PLoS ONE.

[3] Jin Z., Wang C., Yu S., Zhang S., Ding X. (2022). Spatiotemporal evolution and influencing factors of urban shrinkage in the Yellow River Basin, China. PLoS ONE.

[4] Zhang J., Shang Y., Cui M., Luo Q., Zhang R. (2021). Successful and sustainable governance of the lower Yellow River, China: A floodplain utilization approach for balancing ecological conservation and development. Environ. Dev. Sustain..

[5] Xu J.-J., Wang H.-J., Tang K. (2022). The sustainability of industrial structure on green eco-efficiency in the Yellow River Basin. Econ. Anal. Policy.

[6] Chen Y.-P., Fu B.-J., Zhao Y., Wang K.-B., Zhao M.M., Ma J.-F., Wu J.-H., Xu C., Liu W.-G., Wang H. (2020). Sustainable development in the Yellow River Basin: Issues and strategies. J. Clean. Prod..

[7] Yang S., Hao H., Liu B., Wang Y., Yang Y., Liang R., Li K. (2021). Influence of socioeconomic development on river water quality: A case study of two river basins in China. Environ. Sci. Pollut. Res..

[8] Ramanathan R., He Q., Black A., Ghobadian A., Gallear D. (2017). Environmental regulations, innovation and firm performance: A revisit of the Porter hypothesis. J. Clean. Prod..

[9] Wang Z., Chen Q. (2022). Comprehensive partitions and optimisation strategies based on tourism urbanisation and resources environment carrying capacity in the Yellow River Basin, China. Environ. Sci. Pollut. Res..

[10] Ulucak R., Khan SU D., Baloch M.A., Li N. (2019). Mitigation pathways toward sustainable development: Is there any trade-off between environmental regulation and carbon emissions reduction?. Sustain. Dev..

[11] Lanoie P., Patry M., Lajeunesse R. (2008). Environmental regulation and productivity: Testing the porter hypothesis. J. Product. Anal..

[12] Chen Y., Fan X., Zhou Q. (2020). An Inverted-U Impact of Environmental Regulations on Carbon Emissions in China’s Iron and Steel Industry: Mechanisms of Synergy and Innovation Effects. Sustainability.

[13] Féres J., Reynaud A. (2011). Assessing the Impact of Formal and Informal Regulations on Environmental and Economic Performance of Brazilian Manufacturing Firms. Environ. Resour. Econ..

[14] Pargal S., Wheeler D. (1996). Informal Regulation of Industrial Pollution in Developing Countries: Evidence from Indonesia. J. Polit. Econ..

[15] Dibben P., Wood G., Williams C.C. (2015). Pressures towards and against formalization: Regulation and informal employment in Mozambique. Int. Labour Rev..

[16] Wang L., Wang Z., Ma Y. (2022). Heterogeneous environmental regulation and industrial structure upgrading: Evidence from China. Environ. Sci. Pollut. Res..

[17] Ramanathan R., Black A., Nath P., Muyldermans L. (2010). Impact of environmental regulations on innovation and performance in the UK industrial sector. Manag. Decis..

[18] Liu W., Tong J., Yue X. (2016). How Does Environmental Regulation Affect Industrial Transformation? A Study Based on the Methodology of Policy Simulation. Math. Probl. Eng..

[19] Porter M.E., van der Linde C. (1995). Toward a New Conception of the Environment-Competitiveness Relationship. J. Econ. Perspect..

[20] Lanoie P., Laurent-Lucchetti J., Johnstone N., Ambec S. (2011). Environmental Policy, Innovation and Performance: New Insights on the Porter Hypothesis. J. Econ. Manag. Strat..

[21] Yu X., Wang P. (2021). Economic effects analysis of environmental regulation policy in the process of industrial structure upgrading: Evidence from Chinese provincial panel data. Sci. Total. Environ..

[22] Stavropoulos S., Wall R., Xu Y. (2017). Environmental regulations and industrial competitiveness: Evidence from China. Appl. Econ..

[23] Guan S., Liu J., Liu Y., Du M. (2022). The Nonlinear Influence of Environmental Regulation on the Transformation and Upgrading of Industrial Structure. Int. J. Environ. Res. Public Health.

[24] Zhang X., Li Y., Shi K., Feng Y. (2022). How Do Environmental Technology Standards Affect the Green Transformation? New Evidence from China. Int. J. Environ. Res. Public Health.

[25] Hu W., Xiong Z. (2021). Do stringent environmental regulations help improve the total factor carbon productivity? Empirical evidence from China’s industrial sectors. Appl. Econ..

[26] Yu B., Lu L. (2022). Regional Environmental Regulation and Industrial Structure Upgrading: Prefecture-Level Evidence from China. J. Environ. Sci. Manag..

[27] Liu C., Tang C., Liu Z., Huang Y. (2022). How does public environmental supervision affect the industrial structure optimization?. Environ. Sci. Pollut. Res. Int..

[28] Xu J., Chen D., Liu R., Zhou M., Kong Y. (2021). Environmental Regulation, Technological Innovation, and Industrial Transformation: An Empirical Study Based on City Function in China. Sustainability.

[29] Zhang Z., Chen H. (2022). Dynamic interaction of renewable energy technological innovation, environmental regulation intensity and carbon pressure: Evidence from China. Renew. Energy.

[30] Sun J., Tang D., Kong H., Boamah V. (2022). Impact of Industrial Structure Upgrading on Green Total Factor Productivity in the Yangtze River Economic Belt. Int. J. Environ. Res. Public Health.

[31] Dong X., Zhong Y., Liu M., Xiao W., Qin C. (2022). Research on the impacts of dual environmental regulation on regional carbon emissions under the goal of carbon neutrality-the intermediary role of green technology innovation. Front. Environ. Sci..

[32] Yuan B., Xiang Q. (2018). Environmental regulation, industrial innovation and green development of Chinese manufacturing: Based on an extended CDM model. J. Clean. Prod..

[33] Song M., Tao W., Shen Z. (2022). Improving high-quality development with environmental regulation and industrial structure in China. J. Clean. Prod..

[34] Kneller R., Manderson E. (2012). Environmental regulations and innovation activity in UK manufacturing industries. Resour. Energy Econ..

[35] Van der Heijden J. (2020). Environmental regulation in the twenty-first century: A systematic review of (and critical research agenda for) JEPP scholarship. J. Environ. Policy Plan..

[36] Ardiwinata J.S., Zaman K., Nassani A.A., Haffar M., Pasani C.F., Sriyanto S. (2022). What is Right and What is Wrong in the Environmental Governance Model? Environmental Regulations for Improving Environmental Sustainability Ratings. Probl. Ekorozwoju.

[37] Cui S., Wang Y., Zhu Z., Zhu Z., Yu C. (2022). The impact of heterogeneous environmental regulation on the energy eco-efficiency of China’s energy-mineral cities. J. Clean. Prod..

[38] Phuong P.T., Mol A. (2004). Communities as informal regulators: New arrangements in industrial pollution control in Viet Nam. J. Risk Res..

[39] Khan M., Chaudhry M.N., Ahmad S.R., Saif S. (2019). The role of and challenges facing non-governmental organizations in the environmental impact assessment process in Punjab, Pakistan. Impact Assess. Proj. Apprais..

[40] Xiong B., Wang R. (2020). Effect of Environmental Regulation on Industrial Solid Waste Pollution in China: From the Perspective of Formal Environmental Regulation and Informal Environmental Regulation. Int. J. Environ. Res. Public Health.

[41] Sinn H.-W. (2008). Public policies against global warming: A supply side approach. Int. Tax Public Finance.

[42] Dasgupta S., Mody A., Roy S., Wheeler D. (2001). Environmental Regulation and Development: A Cross-Country Empirical Analysis. Oxf. Dev. Stud..

[43] Wang X., Shao Q. (2019). Non-linear effects of heterogeneous environmental regulations on green growth in G20 countries: Evidence from panel threshold regression. Sci. Total Environ..

[44] Shen T., Chen H.H., Zhao D.H., Qiao S. (2022). Examining the impact of environment regulatory and resource endowment on technology innovation efficiency: From the microdata of Chinese renewable energy enterprises. Energy Rep..

[45] Borsatto J., Bazani C.L. (2021). Green innovation and environmental regulations: A systematic review of international academic works. Environ. Sci. Pollut. Res..

[46] Murshed M., Rahman A., Alam S., Ahmad P., Dagar V. (2021). The nexus between environmental regulations, economic growth, and environmental sustainability: Linking environmental patents to ecological footprint reduction in South Asia. Environ. Sci. Pollut. Res..

[47] Chen L., Ye W., Huo C., James K. (2020). Environmental Regulations, the Industrial Structure, and High-Quality Regional Economic Development: Evidence from China. Land.

[48] Behera P., Sethi N. (2022). Nexus between environment regulation, FDI, and green technology innovation in OECD countries. Environ. Sci. Pollut. Res..

[49] Pan X., Ai B., Li C., Pan X., Yan Y. (2019). Dynamic relationship among environmental regulation, technological innovation and energy efficiency based on large scale provincial panel data in China. Technol. Forecast. Soc. Change.

[50] Zhou Q., Shi M., Huang Q., Shi T. (2021). Do Double-Edged Swords Cut Both Ways? The Role of Technology Innovation and Resource Consumption in Environmental Regulation and Economic Performance. Int. J. Environ. Res. Public Health.

[51] Dechezleprêtre A., Sato M. (2017). The Impacts of Environmental Regulations on Competitiveness. Rev. Environ. Econ. Policy.

[52] Wang G., Liu S. (2020). Is technological innovation the effective way to achieve the “double dividend” of environmental protection and industrial upgrading?. Environ. Sci. Pollut. Res. Int..

[53] Du K., Cheng Y., Yao X. (2021). Environmental regulation, green technology innovation, and industrial structure upgrading: The road to the green transformation of Chinese cities. Energy Econ..

[54] Liu X., Chen S. (2022). Has environmental regulation facilitated the green transformation of the marine industry?. Mar. Policy.

[55] Chen L., Li W., Yuan K., Zhang X. (2021). Can informal environmental regulation promote industrial structure upgrading? Evidence from China. Appl. Econ..

[56] Zhang H., Xu T., Feng C. (2022). Does public participation promote environmental efficiency? Evidence from a quasi-natural experiment of environmental information disclosure in China. Energy Econ..

[57] Jiang Z., Lyu P. (2021). Stimulate or inhibit? Multiple environmental regulations and pollution-intensive Industries’ Transfer in China. J. Clean. Prod..

[58] Zhang Z., Zhang G., Song S., Su B. (2020). Spatial Heterogeneity Influences of Environmental Control and Informal Regulation on Air Pollutant Emissions in China. Int. J. Environ. Res. Public Health.

[59] Jamalpuria A. (2013). On information dissemination as an informal environmental regulation. Environ. Dev. Econ..

[60] Abbas S., Gui P., Chen A., Ali N. (2022). The effect of renewable energy development, market regulation, and environmental innovation on CO2 emissions in BRICS countries. Environ. Sci. Pollut. Res..

[61] Chen X., Chen Y.E., Chang C.-P. (2019). The effects of environmental regulation and industrial structure on carbon dioxide emission: A non-linear investigation. Environ. Sci. Pollut. Res..

[62] Yin J., Zheng M., Chen J. (2015). The effects of environmental regulation and technical progress on CO2 Kuznets curve: An evidence from China. Energy Policy.

[63] Kathuria V. (2007). Informal regulation of pollution in a developing country: Evidence from India. Ecol. Econ..

[64] Feng Z., Chen W. (2018). Environmental Regulation, Green Innovation, and Industrial Green Development: An Empirical Analysis Based on the Spatial Durbin Model. Sustainability.

[65] Jia L., Hu X., Zhao Z., He B., Liu W. (2022). How Environmental Regulation, Digital Development and Technological Innovation Affect China’s Green Economy Performance: Evidence from Dynamic Thresholds and System GMM Panel Data Approaches. Energies.

[66] Wang G., Cheng K., Luo Y., Salman M. (2022). Heterogeneous environmental regulations and green economic efficiency in China: The mediating role of industrial structure. Environ. Sci. Pollut. Res..

[67] Zheng H., Zhang L., Song W., Mu H. (2022). Pollution heaven or pollution halo? Assessing the role of heterogeneous environmental regulation in the impact of foreign direct investment on green economic efficiency. Environ. Sci. Pollut. Res..

[68] Wang Z., Wang N., Hu X., Wang H. (2022). Threshold effects of environmental regulation types on green investment by heavily polluting enterprises. Environ. Sci. Eur..

[69] Khattak S.I., Khan A.M., Khan M.K., Li C., Liu J., Pi Z. (2022). Do regional government green innovation preferences promote industrial structure upgradation in China? Econometric assessment based on the environmental regulation threshold effect model. Front. Environ. Sci..

[70] Wang B., Ma C., Wu J. (2022). Does central environmental inspection promote the industrial structure upgrading in China? An attention-based view. Front. Environ. Sci..

[71] Wei H., Yao H. (2022). Environmental Regulation, Roundabout Production, and Industrial Structure Transformation and Upgrading: Evidence from China. Sustainability.

[72] Xie R., Teo T.S.H. (2022). Green technology innovation, environmental externality, and the cleaner upgrading of industrial structure in China—Considering the moderating effect of environmental regulation. Technol. Forecast. Soc. Change.

[73] Li F., Fang H., Deng S. (2022). Environmental Regulation, Industrial Structure Upgrading and Technological Innovation: Empirical Data from Guangdong Province in China. Pol. J. Environ. Stud..

[74] Yang Y., Tang D., Zhang P. (2020). Double Effects of Environmental Regulation on Carbon Emissions in China: Empirical Research Based on Spatial Econometric Model. Discret. Dyn. Nat. Soc..

[75] Zhang H., Liu Z., Zhang Y.-J. (2022). Assessing the economic and environmental effects of environmental regulation in China: The dynamic and spatial perspectives. J. Clean. Prod..

